# Using machine learning methods to investigate the role of volatile organic compounds in non-alcoholic fatty liver disease

**DOI:** 10.3389/fmolb.2025.1631265

**Published:** 2025-08-06

**Authors:** Chih-Hao Shen, Ruei-Hao Huang, Yaw-Kuen Li, Ta-Wei Chu, Dee Pei

**Affiliations:** ^1^ Division of Pulmonary and Critical Care Medicine, Department of Medicine, Tri-Service General Hospital, National Defense Medical Center, Taipei, Taiwan; ^2^ Center for Emergent Functional Matter Science, National Yang Ming Chiao Tung University, Hsinchu, Taiwan; ^3^ Department of Obstetrics and Gynecology, Tri-Service General Hospital, National Defense Medical Center, Taipei, Taiwan; ^4^ Department CEO, MJ Health Research Foundation, Taipei, Taiwan; ^5^ Department of Medicine, Medical School, Fu Jen Catholic University, New Taipei City, Taiwan; ^6^ Division of Endocrinology and Metabolism, Department of Internal Medicine, School of Medicine, College of Medicine, Fu Jen Catholic University Hospital, New Taipei City, Taiwan

**Keywords:** Volatile Organic Compounds, non-alcoholic fatty liver, machine learning, AI, cohort

## Abstract

**Aims:**

Approximately 25%–30% of the global population is affected by non-alcoholic fatty liver disease (NAFLD). This study aimed to explore whether NAFLD could be effectively detected using 341 volatile organic compounds (VOCs) via 10 machine learning (Mach-L) algorithms in a cohort of 1,501 individuals.

**Methods:**

Participants were selected from the Taiwan MJ cohort, which includes comprehensive demographic, biochemical, lifestyle, and VOCs data. NAFLD was diagnosed by experienced gastroenterologists. Exhaled breath samples were collected using a 1.0-L aluminum bag (late expiratory fraction) and analyzed with selected-ion flow-tube mass spectrometry. Ten Mach-L techniques were employed to evaluate two predictive models: Model 1 (demographic, lifestyle, and biochemical data), and Model 2 (Model 1 + VOCs), assessed using area under the receiver operating characteristic curve (AUC).

**Results:**

Subjects with NAFLD had significantly higher values for age, BMI, blood pressure, and other biomedical markers, except for eGFR and HDL-C. Key predictors of NAFLD included BMI, triglycerides (TG), uric acid (UA), fasting plasma glucose (FPG), γ-GT, gender, LDL-C, and sleep duration. The addition of VOCs to Model 1 improved the AUC from 0.722 ± 0.149 to 0.770 ± 0.264 (p < 0.001). Ten VOCs were identified as the most influential, in order of importance: 2-propanol, acetone, butyl 2-methylbutanoate, diethylethanolamine, urethane, β-caryophyllene, furfural, tridecane, 4-methyloctanoic acid, and (S)-2-methyl-1-butanol.

**Conclusion:**

Incorporating VOCs into traditional demographic, biochemical, and lifestyle data significantly enhanced the model’s predictive performance. This suggests that VOCs may be associated with the underlying pathophysiology of NAFLD.

## Introduction

Non-alcoholic fatty liver disease (NAFLD) is defined as the presence of macrovesicular steatosis in more than 5% of hepatocytes without other identifiable causes, such as alcohol consumption or medication use. NAFLD progresses from simple steatosis to non-alcoholic steatohepatitis, fibrosis, and eventually cirrhosis, making it one of the leading causes of chronic liver disease worldwide (Younossi et al., 2016; Ghevariya et al., 2014). The global prevalence of NAFLD has increased from 15% in 2005 to 25%–30% in 2023, reflecting the global rise in obesity rates (Quek et al., 2023). In Taiwan, a similar trend has been observed, with two studies estimating that 11.4%–41% of the general population may be affected by NAFLD (Chen et al., 2006; Lin et al., 2005). Consequently, early detection and prevention of NAFLD have become key priorities for healthcare providers and policymakers.

Traditionally, multiple logistic regression (MLR) has been used to analyze the relationship between risk factors and disease outcomes in medical research. The performance of MLR models is commonly evaluated using the area under the receiver operating characteristic curve (AUC). Recently, machine learning (Mach-L)—a branch of artificial intelligence that allows algorithms to learn from past data without explicit programming—has emerged as a competitive and often superior approach to MLR (Marateb et al., 2014; Ye et al., 2020; Nusinovici et al., 2020). Unlike MLR, Mach-L can model complex, nonlinear interactions among multiple variables, making it more suitable for disease prediction tasks (Miller and Brown, 2018). Mach-L in the medical field involves using computer algorithms to analyze large amounts of healthcare data, helping with tasks. These tools can detect patterns in medical images, electronic health records, and other data faster and often more accurately than humans, leading to earlier diagnoses, better patient care, and more efficient healthcare delivery (Arkoudis and Papadakos, 2025).

For over five decades, researchers have shown increasing interest in volatile organic compounds (VOCs) emitted from the human body. In 1971, Nobel laureate Linus Pauling reported that human breath contains approximately 250 VOCs (Machado and Cortez-Pinto, 2014). Later, in 1999, Maurice and Manousou (2018) identified more than 3,400 VOCs in exhaled breath. Alterations in VOC concentrations can reflect disease states, such as cancer (Wei et al., 2020). As a result, breath-derived VOCs have been proposed as biomarkers for detecting metabolic changes associated with various diseases. There have been studies investigated the relationships between VOCs and NAFLD in the past. However, most of these studies focused on how VOCs affect or damage liver. The proposed mechanisms included metabolic dysregulation, oxidative stress, and cell death (Lang and Beier, 2018; Liu et al., 2023; Duan et al., 2025). Their goals were different from the present study. Analytical techniques like gas chromatography-mass spectrometry (GC-MS) have confirmed these associations in numerous studies (Samudrala et al., 2014; Markar et al., 2019; Ratiu et al., 2020; Keogh and Riches, 2022; Chung et al., 2022). However, while many studies have explored VOC-based disease identification, few have utilized Mach-L techniques for VOC profiling (Tsou et al., 2021; Shaffie et al., 2022; Sukaram et al., 2023).

In this study, we employed 10 different Mach-L algorithms to develop predictive models for NAFLD using health examination data combined with exhaled VOC profiles. The performance of these models was compared to evaluate their potential utility in clinical screening for NAFLD. Finally, by applying Shapley addictive explanation to examine the directions and strengths of impacts.

## Materials and methods

This study utilized data from the ongoing Taiwan MJ cohort, a prospective cohort collected through health examinations conducted by the MJ Health Screening Centers in Taiwan (Wu et al., 2017). The dataset includes over 100 essential biological indicators such as anthropometric measurements, blood tests, and imaging tests, among others.

The data were obtained from MJ clinic. At the time of their health checkups, participants provided general consent forms for future anonymous research. This database was maintained by the Interpretation Foundation of MJ Health Research Foundation. All or part of the data used in this study were authorized and provided by the foundation (Authorization Code: MJHRF2022009A). However, it is important to note that all interpretations and conclusions in this study are those of the authors and do not necessarily represent the views of the MJ Health Research Foundation.

The study protocol was reviewed and approved by the Institutional Review Board of National Yang Ming Chiao Tung University, Taiwan (IRB No. NCTU-REC-109-074E). All participants signed a written informed consent form after receiving a thorough explanation of the study’s purpose, procedures, and potential risks by trained research assistants. These assistants ensured that all explanations were delivered using clear and understandable language, allowing participants to fully comprehend the study. After ample time for questions and deliberation, participants who provided informed and voluntary consent signed the consent form.

A total of 2,152 participants who underwent both medical ultrasound diagnosis for NAFLD and three sessions of exhaled breath volatile organic compounds (VOCs) collection (a total of 6,363 records) were included initially.

The inclusion criteria are:

1. Subjects between 30-70
2. With data of VOCs

Our exclusion criteria are:

1. Having significant medical diseases such as myocardial infarction, stroke, or cancers
2. Having drinking alcohol habit
3. Miss important data such as age, body mass index (BMI) or blood pressure

**FIGURE 1:**
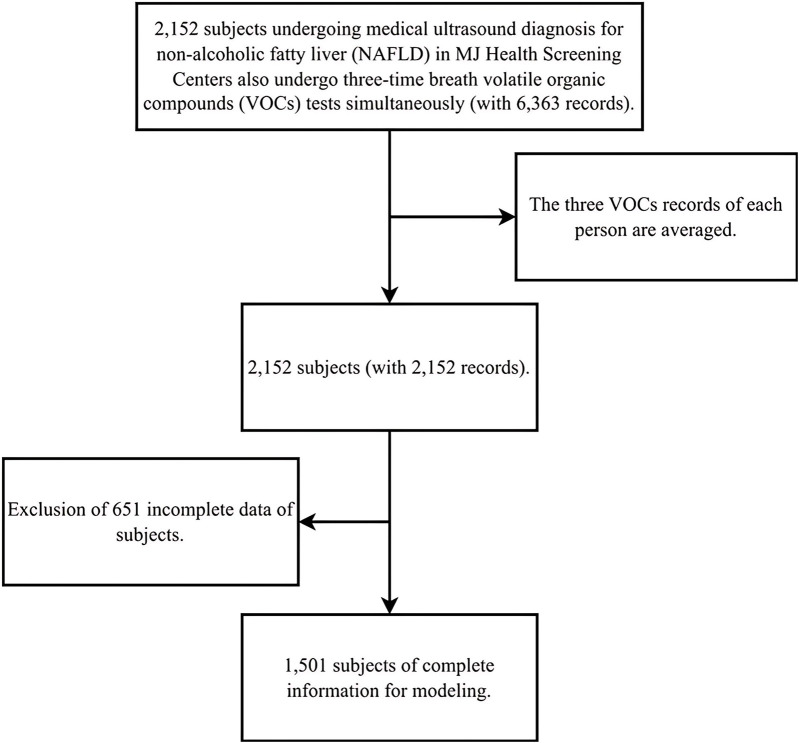
The participants selection scheme.

After excluding 651 records due to data loss or specific conditions, the final analysis included 1,501 individuals, as shown in Figure 1.

### Clinical assessments and biochemical analyses

Details of obtaining basic parameters such as BMI, blood pressure, collecting blood samples, and questionnaires could be referred to our previous publication.

#### Fatty liver diagnosis

The diagnosis of fatty liver was based on ultrasound features, including increased hepatic parenchymal brightness, liver-to-kidney contrast, deep beam attenuation, visible intrahepatic vessels, and gallbladder wall definition. Qualitative grading classified fatty liver into mild, moderate, or severe, corresponding to grades 1 to 3, respectively, with grade 0 representing a normal liver (Mahale et al., 2018; Dasarathy et al., 2009). For the purpose of this study, grades 1–3 were collectively defined as having fatty liver (NAFLD).

#### Variable selection

Seventeen clinical variables potentially associated with NAFLD were selected (listed in Table 1) as independent variables. NAFLD status (yes/no) was used as the dependent categorical variable.

**TABLE 1:** The demographic, biochemistry, and volatile organic compounds data of the study cohort.

	Men	Women
N	653	848
Age (year)	42 ± 12	46 ± 12
Body mass index (kg/m^2^)	21.3 ± 2.52	24.9 ± 3.84***
Systolic blood pressure (mmHg)	111.2 ± 15.9	119.8 ± 16.9***
Diastolic blood pressure (mmHg)	72.1 ± 10.3	77.4 ± 11.1***
Fasting plasma glucose (mg/dL)	96.5 ± 9.36	104.0 ± 17.0***
Total bilirubin (mg/dL)	1.09 ± 0.388	1.04 ± 0.401*
Albumin (g/dL)	4.40 ± 0.222	4.43 ± 0.229***
Alkaline phosphatase (U/L)	56.7 ± 16.3	64.3 ± 18.9***
Serum glutamic oxaloacetic transaminase (U/L)	21.4 ± 6.97	24.7 ± 14.6***
Serum glutamic pyruvic transaminase (U/L)	20.8 ± 13.2	32.1 ± 29.4***
γ-Glutamyl transferase (U/L)	19.5 ± 14.9	31.6 ± 33.6***
Estimated glomerular filtration rate (ml/min/1.73 m2)	86.1 ± 13.2	83.3 ± 13.7***
Uric acid (mg/dL)	5.17 ± 1.17	6.02 ± 1.43***
Triglyceride (mg/dL)	69.1 ± 33.8	115.6 ± 74.2***
High density lipoprotein cholesterol (mg/dL)	58.2 ± 16.4	57.6 ± 15.0
Low density lipoprotein cholesterol (mg/dL)	110.9 ± 28.7	126.9 ± 35.3***
Alpha-fetoprotein (ng/mL)	2.78 ± 1.91	3.05 ± 1.86**
Beta-caryophyllene (87-44-5)	0.020 ± 0.148	0.010 ± 0.102
Furfural (98-01-1)	5.05 ± 15.4	4.69 ± 9.11
Tridecane (629-50-5)	1.08 ± 3.62	1.89 ± 7.48*
Butyl 2-methylbutanoate (15706-73-7)	0.134 ± 0.521	0.133 ± 0.683
Diethylethanolamine (100-37-8)	3.03 ± 9.19	2.44 ± 5.72
4-methyloctanoic acid (54947-74-9)	0.170 ± 0.743	0.119 ± 0.735
(S)-2-methyl-1-butanol (1565-80-6)	4.89 ± 37.5	3.85 ± 8.74
Urethane (51-79-6)	20.7 ± 64.1	18.2 ± 29.3
2-propanol (67-63-0)	71.4 ± 161.1	61.2 ± 205.4
Acetone (67-64-1)	1,221.1 ± 3,008.5	1,053.7 ± 4,885.6

Smoking	n (%)	
Non-smoker	1140 (75)	
Less than 10 cigarettes/week	59 (3.9)	
10–20 cigarettes/week	129 (8.6)	
20 cigarettes/week	54 (3.4)	
More than 20 cigarettes/week	119 (7.6)	

Sleeping	n (%)	
Less than 6 h/day	17 (4.7)	
6 h/day	375 (25)	
7 h/day	713 (47.5)	
8 h/day	347 (24.9)	
9 h/day	39 (2.6)	
More than 9 h/day	10 (7)	

### Protocol for breath sample collection

All volunteer participants remained in a designated room under resting conditions for at least 10 min prior to sample collection. To minimize contamination, each participant was asked to rinse their mouth with unchlorinated water before exhaling through a mouthpiece connected to a three-way direct-connect valve.

Initially, exhaled breath passed through the first outlet, which was connected to a gas bag (SKC Inc., Eighty-Four, PA, United States) to estimate the volume of exhaled air. Once the volume of the initial exhalation reached approximately 0.3 L, the valve was switched to the second outlet, which was attached to a 1.0-L aluminum bag. This second bag was used to collect the late expiratory fraction, which is more representative of alveolar air and thus suitable for volatile organic compound (VOC) analysis.

To ensure adequate sample volume for analysis, the collection procedure was repeated two to three times as necessary. All collected breath samples were sealed, stored at room temperature (25°C), and analyzed within 48 h.

To validate the stability of VOCs under these storage conditions, a time-dependent analysis was conducted on ten breath samples. Samples were analyzed twice daily over three consecutive days. Comparison of the quantitative VOC data indicated that the majority of compounds remained stable during the storage period.

#### VOCs analysis using SIFT-MS

A selected-ion flow-tube mass spectrometry system (SIFT-MS; VOICE200 Ultra, Syft Technologies, Christchurch, New Zealand) was employed to analyze volatile organic compounds (VOCs) in the collected late expiratory breath fraction. It is a quantitative mass spectrometry technique used for real-time analysis of trace volatile compounds, especially volatile organic compounds (VOCs), in air, breath, or headspace above liquids without the need for sample preparation or chromatographic separation (Španěl and Smith, 2020).

In this method, selected precursor ions (H_3_O^+^, NO^+^, and O_2_^+^) are injected into a nitrogen carrier gas within the flow tube. When breath samples are introduced, VOCs present in the sample undergo ionization, resulting in the formation of characteristic product ions. These product ions are detected by a quadrupole mass spectrometer, which measures the count rates of both precursor and product ions in real time.

For VOCs with significant product ion overlap that could not be resolved using the tolerance setting, concentrations were reported on a relative scale. Statistical models were constructed based on both absolute concentrations and these relative measures to ensure robustness in VOC profiling and interpretation.

To assure the reproducibility and data reliability we standardized and calibrated with the following methods:

1. Traceable reference materials: Use primary standards (e.g., NIST-traceable mixtures) for instrument calibration and secondary/working standards for routine checks (RI-URBANS, 2024; Dusanter et al., 2025).
2. Matrix modifiers: Add salt solutions (e.g., NaCl) to normalize partitioning behavior of VOCs in complex samples, reducing bias from dissolved solutes or organic components (U.S. Environmental Protection Agency, 2014; Final Report, 2012).
3. Dynamic calibration: For instruments like PTR-MS, use gas standards with known VOC concentrations and proton transfer rate constants to calculate normalized sensitivities (Dusanter et al., 2025).

### Machine learning-based analysis technology

While numerous studies have explored the application of VOC measurements for disease identification (Ratiu et al., 2020; Keogh and Riches, 2022; Chung et al., 2022), relatively few have focused on utilizing Mach-L techniques specifically for VOC profiling (Tsou et al., 2021; Shaffie et al., 2022; Sukaram et al., 2023). In this study, we employed ten distinct Mach-L algorithms to construct predictive models for diagnosing non-alcoholic fatty liver disease (NAFLD) based on VOCs collected from exhaled breath. To assess the impact of VOCs, models were developed both with and without VOC data, and their predictive performances were compared.

The ten machine learning techniques applied are as follows:

•Random Forest (RF): An ensemble learning method utilizing multiple unpruned decision trees for classification (Breiman, 2001).
• C5.0 Decision Trees (C5.0): A rule-based model using entropy, information gain, and gain ratio for decision tree construction (Quinlan, 2004).
•Stochastic Gradient Boosting (SGB): Combines bagging and boosting to construct additive regression tree models (Friedman, 2001).
•Multivariate Adaptive Regression Splines (MARS): A non-parametric regression technique using piecewise polynomial functions (Friedman, 1991).
•Classification and Regression Tree (CART): A decision tree model built using Gini impurity for splitting nodes (Breiman et al., 1984).
•Least Absolute Shrinkage and Selection Operator (Lasso): A linear model applying L1 regularization to perform feature selection (Hastie et al., 2015).
•Ridge Regression (Ridge): Similar to Lasso but uses L2 regularization for coefficient shrinkage (Hoerl and Kennard, 2000).
•Extreme Gradient Boosting (XGBoost): An optimized gradient boosting algorithm designed for speed and performance (Meng et al., 2016).
•Gradient Boosting with Categorical Features (CatBoost): A boosting technique optimized for categorical features using an ordered boosting method (Dorogush et al., 2018).
•Light Gradient Boosting Machine (LightGBM): A fast, histogram-based gradient boosting algorithm designed for efficiency and scalability (Ke et al., 2017).

Although Mach-L algorithms are capable of identifying key predictor variables, relying on a single method may lead to suboptimal and biased feature selection. To overcome this limitation, variable ensemble strategies are often employed, which integrate the outputs from multiple algorithms. Prior research indicates that such ensemble approaches enhance variable selection robustness, reducing both bias and variance (Pes, 2020; Moghimi et al., 2018; Tuli et al., 2019).

In this study, the variable importance values generated by each Mach-L model were averaged. The top 10 VOCs, ranked by average importance across all models, were selected for further discussion.

All analyses were conducted using the R programming language (version 4.1.2, R Core Team, Vienna, Austria) and RStudio (version 1.1.453) (R Core Team, 2017; RStudio Team, 2015). The following R packages were employed for model development: random Forest, C50, gbm, RWeka, kernlab, earth, rpart, glmnet, XGBoost, LightGBM, and cat boost. Heatmaps were visualized using the pheatmap package (version 2.6.2) (Browne, 2000; Gu, 2022).

To train and evaluate each Mach-L model, an 80/20 train-test split was used. The training set (80%) was used to construct models, while the testing set (20%) evaluated predictive performance. Hyperparameter tuning was conducted using 10-fold cross-validation (CV) to ensure optimal performance for each algorithm. The final model for each method was selected based on the best-performing configuration. Cross-validation procedures were executed using the caret package (version 6.0-93) (Kuhn, 2022).

In order to understand the directions and impacts of the variables, XGboost SHAP was applied using the following Python packages: SHAP, the core package for computing and visualizing SHAP values, provides interpretability for model predictions and feature importance. Pandas, a powerful library for data manipulation and preprocessing, was used to manage datasets, clean data, and prepare inputs for SHAP analysis. NumPy, a fundamental package for numerical computations, supported array operations and numerical calculations required by SHAP. Matplotlib, a plotting library for creating static, interactive, and animated visualizations, was employed to generate SHAP plots, including summary plots, bar plots, and waterfall plots.te feature contributions to specific predictions.

### Performance evaluation metrics

To comprehensively evaluate the predictive performance of the Mach-L, we employed a range of widely accepted performance measures, as recommended in previous studies (Dias Canedo and Cordeiro Mendes, 2020; Hussain et al., 2021; Tomer and Sharma, 2022). Specifically, the following metrics were utilized in our analysis: accuracy (ACC), sensitivity (Sens), and specificity (Spec). These metrics provide an overall understanding of the model’s classification capabilities.

However, when dealing with imbalanced datasets, traditional metrics such as ACC, Sens, and Spec can be misleading, as they tend to be disproportionately influenced by the majority class distribution. To mitigate this issue, we additionally calculated balanced accuracy (BA) and area under the receiver operating characteristic curve (AUC)—both of which are considered more robust and reliable indicators for evaluating model performance under class imbalance conditions (di Biase et al., 2020; Hashim et al., 2021).

•Balanced Accuracy (BA) accounts for imbalanced data by averaging sensitivity and specificity.
•AUC provides a threshold-independent measure of a model’s ability to distinguish between classes.

The definitions and formulas for all performance metrics used in this study are detailed in (Tharwat, 2021).

To assess the impact of volatile organic compounds (VOCs) on model performance, we compared each Mach-L model’s predictive ability with and without VOC features. We applied DeLong’s test for pairwise comparison of AUC values between these two scenarios across all models (DeLong et al., 1988), allowing for a statistically grounded evaluation of VOCs’ contribution to predictive improvement.

## Results

A total of 1,501 participants were included in the present study. Table 1 presents the demographic and clinical characteristics of the participants, stratified by the presence or absence of non-alcoholic fatty liver disease (NAFLD).

As expected, participants diagnosed with NAFLD exhibited significantly higher values across several variables, including age, body mass index (BMI), blood pressure, and various biochemical markers, compared to those without NAFLD. The only exceptions were estimated glomerular filtration rate (eGFR) and high-density lipoprotein cholesterol (HDL-C), which did not follow the same trend.

Among all examined variables, the most influential predictors for identifying NAFLD were found to be: BMI, Triglycerides (TG), Uric acid (UA), Fasting plasma glucose (FPG), Gamma-glutamyl transferase (GGT), Gender, Low-density lipoprotein cholesterol (LDL-C) and Sleeping hours.

In parallel, VOC profiling using 10 different machine learning (Mach-L) techniques identified 10 key VOCs as significant predictors for NAFLD. Ranked from most to least important, these compounds are: 2-Propanol, Acetone, Butyl 2-methylbutanoate, Diethylethanolamine, Urethane, β -Caryophyllene, Furfural, Tridecane, 4-Methyloctanoic acid and (S)-2-Methyl-1-butanol.

Table 2 displays the comparative concentrations of these 10 VOCs in subjects with and without NAFLD, along with their corresponding rankings based on variable importance across the Mach-L models.

**TABLE 2:** t-test comparing volatile organic compounds in subjects with and without NAFLD.

Characteristic (mean ± SD)	NAFLD	Control groups	P value
n (%)	848 (56.50%)	653 (43.50%)	
2-propanol	61.28 ± 205.46	71.42 ± 161.10	0.284
Acetone	1053.72 ± 4885.64	1221.07 ± 3008.48	0.414
butyl 2-methylbutanoate	0.13 ± 0.68	0.13 ± 0.52	0.955
diethylethanolamine	2.45 ± 5.73	3.03 ± 9.20	0.158
urethane	18.24 ± 29.31	20.72 ± 64.05	0.360
beta-caryophyllene	0.01 ± 0.10	0.02 ± 0.15	0.296
furfural	4.69 ± 9.11	5.05 ± 15.38	0.599
tridecane	1.89 ± 7.48	1.08 ± 3.62	0.006
4-methyloctanoic acid	0.12 ± 0.74	0.17 ± 0.74	0.190
(S)-2-methyl-1-butanol	3.85 ± 8.75	4.90 ± 37.47	0.485
methanamide	10.32 ± 16.59	12.21 ± 74.70	0.528
1-nonene	6.51 ± 16.03	5.23 ± 8.66	0.049
isobutane	160.08 ± 392.34	165.93 ± 510.95	0.808
trimethylamine	87.84 ± 306.13	104.05 ± 242.21	0.252
6-methyl-5-hepten-2-one	1.92 ± 2.50	1.93 ± 3.19	0.911
pyridine	1.60 ± 2.75	1.49 ± 2.51	0.414
benzoic acid	0.06 ± 0.22	0.08 ± 0.28	0.129
3-buten-2-one	14.13 ± 27.59	15.42 ± 55.93	0.587
propyl acetate	10.98 ± 14.32	15.81 ± 116.80	0.293
propyne	0.97 ± 3.55	0.68 ± 1.77	0.035

Data are presented as means ± standard deviation (SD) or numbers (%) as in the case; P values of excess statistically significant are from the t-test comparing subjects with and without NAFLD. All the statistical tests of independence were two-sided. Abbreviations: NAFLD, non-alcoholic fatty liver disease.

### Model performance evaluation

The predictive performance of all 10 machine learning (Mach-L) methods is summarized in Table 3. Across all methods, Model 2—which incorporated volatile organic compounds (VOCs)—demonstrated superior performance compared to Model 1, which only included demographic, biochemical, and lifestyle variables. Specifically, accuracy (ACC), sensitivity (Sens), specificity (Spec), BA, and AUC were all improved in Model 2.

**TABLE 3:** Results of machine learning in Model 1 (without VOCs) and Model 2 (with VOCs).

Methods	ACC		Sens		Spec		AUC		BA	
	Model 1	Model 2	Model 1	Model 2	Model 1	Model 2	Model 1	Model 2	Model 1	Model 2
RF	0.734	0.777	0.639	0.858	0.799	0.713	0.777	0.848	0.719	0.785
C5.0	0.691	0.688	0.762	0.843	0.643	0.563	0.683	0.765	0.702	0.703
SGB	0.718	0.784	0.705	0.769	0.726	0.796	0.773	0.854	0.716	0.783
MARS	0.731	0.781	0.680	0.769	0.765	0.790	0.768	0.846	0.723	0.780
CART	0.714	0.781	0.598	0.716	0.793	0.832	0.696	0.781	0.696	0.774
Lasso	0.718	0.787	0.689	0.754	0.737	0.814	0.768	0.866	0.713	0.784
Ridge	0.741	0.744	0.631	0.881	0.816	0.635	0.780	0.832	0.723	0.758
XGBoost	0.688	0.791	0.885	0.828	0.553	0.761	0.784	0.861	0.719	0.794
CatBoost	0.744	0.787	0.697	0.769	0.777	0.802	0.779	0.860	0.737	0.786
LightGBM	0.761	0.787	0.697	0.866	0.805	0.725	0.792	0.860	0.751	0.795

VOC, volatile organic compound; ACC, accuracy; Sens, sensitivity; Spec, specificity; AUC, area under curve; BA, balanced accuracy; Model 1, without VOCs; Model 2, with VOCs; RF, random forest; C5.0, C5.0 decision trees; SGB, stochastic gradient boosting; MARS, multivariate adaptive regression splines; CART, classification and regression tree; Lasso, least absolute shrinkage and selection operator; Ridge, ridge regression; XGBoost, extreme gradient boosting; CatBoost, gradient boosting with categorical features support; LightGBM, light gradient boosting machine.

**FIGURE 2:**
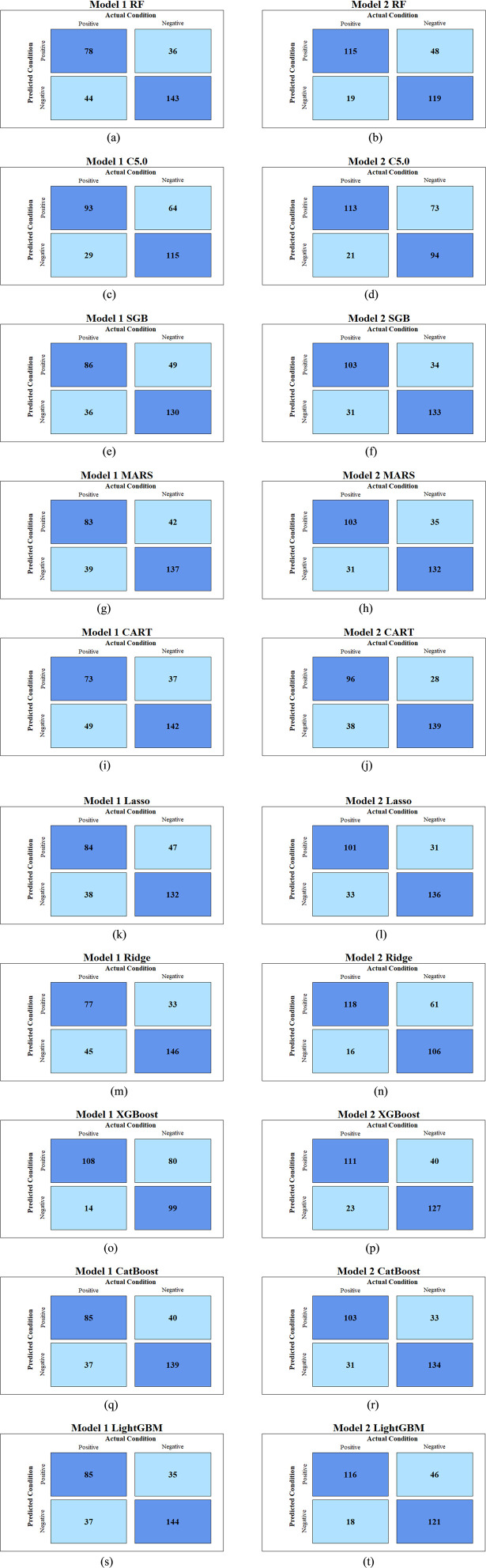
The Confusion matrix of Model 1 and 2 for each machine learning methods. Model 1, without VOCs; Model 2, with VOCs; RF, random forest; C5.0, C5.0 decision trees; SGB, stochastic gradient boosting; MARS, multivariate adaptive regression splines; CART, classification and regression tree; Lasso, least absolute shrinkage and selection operator; Ridge, ridge regression; XGBoost, extreme gradient boosting; CatBoost, gradient boosting with categorical features support; LightGBM, light gradient boosting machine. **(a)** Model 1 o RF. **(b)** Model 2 o RF. **(c)** Model 1 of C5.0. **(d)** Model 2 of C5.0. **(e)** Model 1 of SGB. **(f)** Model 2 of SGB. **(g)** Model 1 of MARS. **(h)** Model 2 of MARS. **(i)** Model 1 of CART. **(j)** Model 2 of CART. **(k)** Model 1 of Lasso. **(l)** Model 2 of Lasso. **(m)** Model 1 of Ridge. **(n)** Model 2 of Ridge. **(o)** Model 1 of XGBoost. **(p)** Model 2 of XGBoost. **(q)** Model 1 of CatBoost. **(r)** Model 2 of CatBoost. **(s)** Model 1 of LightGBM. **(t)** Model 2 of LightGBM.

**FIGURE 3:**
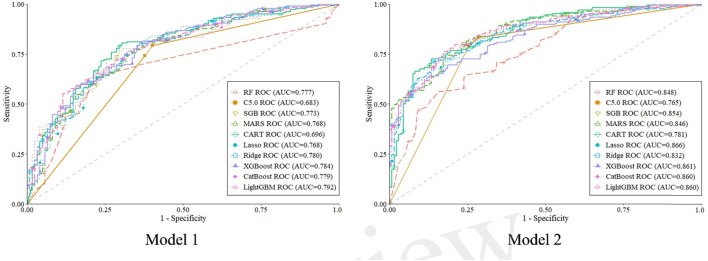
The area under receiver operation curve in model 1 and 2 for all the machine learning methods. Model 1, without VOCs; Model 2, with VOCs; RF, random forest; C5.0, C5.0 decision trees; SGB, stochastic gradient boosting; MARS, multivariate adaptive regression splines; CART, classification and regression tree; Lasso, least absolute shrinkage and selection operator; Ridge, ridge regression; XGBoost, extreme gradient boosting; CatBoost, gradient boosting with categorical features support; LightGBM, light gradient boosting machine.

**FIGURE 4:**
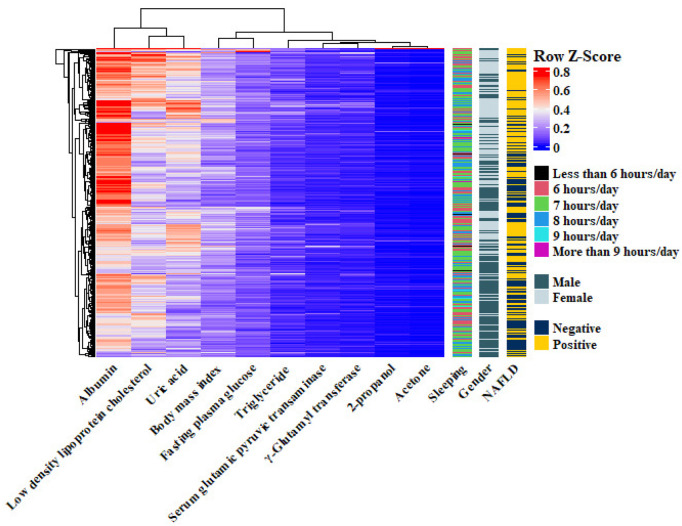
Heatmap of the top 10 volatile organic compounds identified across the machine learning methods.

These findings suggest that the inclusion of VOCs significantly enhanced the predictive accuracy of the models in identifying individuals with NAFLD. The confusion matrices for Models 1 and 2 are presented in Figure 2, while Figure 3 illustrates the respective AUC curves for each model. Additionally, the heatmap of the top 10 VOCs identified across the Mach-L algorithms is shown in Figure 4, highlighting their relative importance in the classification task.

Table 4 presents the pairwise comparisons of AUC values for the 10 Mach-L methods, evaluating the improvement in predictive performance with the inclusion of VOCs compared to models without VOCs. The results indicate that for all methods, the inclusion of VOCs led to a statistically significant improvement in model performance, as evidenced by p-values less than 0.05 across all comparisons. These findings suggest that incorporating VOC data into the Mach-L models for NAFLD diagnosis results in significantly enhanced predictive accuracy compared to models that exclude VOCs.

**TABLE 4:** Pairwise comparisons of the area under curve values between in Model 1 (without VOCs) and Model 2 (with VOCs) using DeLong’s test.

Methods	Model 1	Model 2	Difference Model 1 and Model 2	*p*-value
RF	0.777	0.848	7.10%	−2.050 (0.040)
C5.0	0.683	0.765	8.20%	−1.988 (0.047)
SGB	0.773	0.854	8.10%	−2.384 (0.017)
MARS	0.768	0.846	7.80%	−2.159 (0.031)
CART	0.696	0.781	8.50%	−2.304 (0.021)
Lasso	0.768	0.866	9.80%	−2.846 (0.004)
Ridge	0.780	0.832	5.20%	−1.471 (0.041)
XGBoost	0.784	0.861	7.70%	−2.278 (0.023)
CatBoost	0.779	0.860	8.10%	−2.403 (0.016)
LightGBM	0.792	0.860	6.80%	−2.014 (0.044)

VOC, volatile organic compound; Model 1, without VOCs; Model 2, with VOCs. The numbers in table are the corresponding p-values. *p* < 0.05 was considered statistically significant.

Table 5 displays the most important predictive factors identified by the Mach-L methods, encompassing demographic, biochemical, lifestyle, and VOC-related variables. In total, 25 factors were selected, including the top 10 VOCs. Among the non-VOC variables, BMI emerged as the most influential predictor, followed by triglycerides (TG), uric acid (UA), fasting plasma glucose (FPG), γ -glutamyl transferase (γ -GT), gender, GPT, LDL-cholesterol, sleep duration, albumin, total bilirubin, alkaline phosphatase, GOT, HDL-cholesterol, and diastolic blood pressure (DBP). Notably, beginning from the 10th rank in overall importance, 2-propanol was the first VOC to appear. The complete list of VOCs identified is detailed in the Methods section.

**TABLE 5:** The variables selected and the mean and rank of important values by ten machine learning methods.

Variables	RF	C5.0	SGB	MARS	CART	Lasso	Ridge	XGBoost	Catboost	LightGBM	Mean	ROI
Body mass index (kg/m^2^)	100	100	100	100	100	100	100	100	100	100	100.00	1
Triglyceride (mg/dL)	53.56	100	46.48	52.75	87.96	3.34	2.8	52.45	64.16	33.9	49.74	2
Uric acid (mg/dL)	7.06	32.42	2.27	30.95	39.63	22.12	80	0	1.43	4.42	22.03	3
FPG (mg/dL)	5.61	51.5	3.3	0	0	3.76	8	2.31	7.43	1.18	8.31	4
γ-GT (U/L)	10.34	0	6.35	0	47.7	0	1	6.5	6.72	2.7	8.13	5
Gender	0.07	1.17	0	0	0	0	67	0	0	0	6.82	6
GPT (U/L)	7.62	0	4.22	0	38.8	1.44	4.3	1.57	4.16	1.69	6.38	7
LDL-cholesterol (mg/dL)	5.4	19.75	3.95	14.61	8.33	1.33	2.8	1.31	2.63	2.64	6.28	8
Sleep time	0.74	11.17	0	0	0	0	39	0	0.36	0	5.13	9
2-propanol	1.63	35.92	2.04	0	7.95	0	0	0.43	2.75	0	5.07	10
Albumin (g/dL)	0.97	4.67	0	0	0	0	42	0	0	0	4.76	11
Acetone	1.4	0	0	35.85	0	0	0	0	0.33	1.29	3.89	12
Butyl 2-methylbutanoate	0.26	0	0	0	0	0	32	0	4.31	0	3.66	13
Total bilirubin (mg/dL)	3.35	17.33	2.09	8.83	0	0	0	0	1.86	1.99	3.55	14
AP (U/L)	2.96	22.92	0	0	0	0.08	2.1	0.47	1.07	1.38	3.10	15
Diethylethanolamine	1.21	0	3.73	21.85	0	0	0	0	2.19	1.26	3.02	16
urethane	0.75	4.08	0	24.32	0	0	0	0	0	0	2.92	17
GOT (U/L)	1.3	0.67	0	21.85	0	0	0.11	0	4.42	0.24	2.86	18
HDL-cholesterol (mg/dL)	2.56	17.08	2.09	2.37	0	0	0.93	0	0	0.6	2.56	19
β-caryophyllene	0	0	0	0	0	0	25	0	0	0	2.50	20
DBP (mmHg)	3.19	13.58	0	0	0	1.33	4.8	0	1.33	0.53	2.48	21
Furfural	0.87	22.42	0	0	0	0	0	0	0	0	2.33	22
Tridecane	0.58	15.08	0	0	0	0	4.7	0	2.05	0.07	2.25	23
4-methyloctanoic acid	0.34	21.67	0	0	0	0	0	0	0	0	2.20	24
(S)-2-methyl-1-butanol	0.62	21	0	0	0	0	0	0	0.04	0	2.17	25
…	…	…	…	…	…	…	…	…	…	…	…	…

Abbreviations: ROI, rank of importance; FPG, fasting plasma glucose; γ-GT, γ-glutamyl transferase; GPT, serum glutamic pyruvic transaminase; AP, alkaline phosphatase; GOT, serum glutamic oxaloacetic transaminase; DBP, diastolic blood pressure.

**FIGURE 5:**
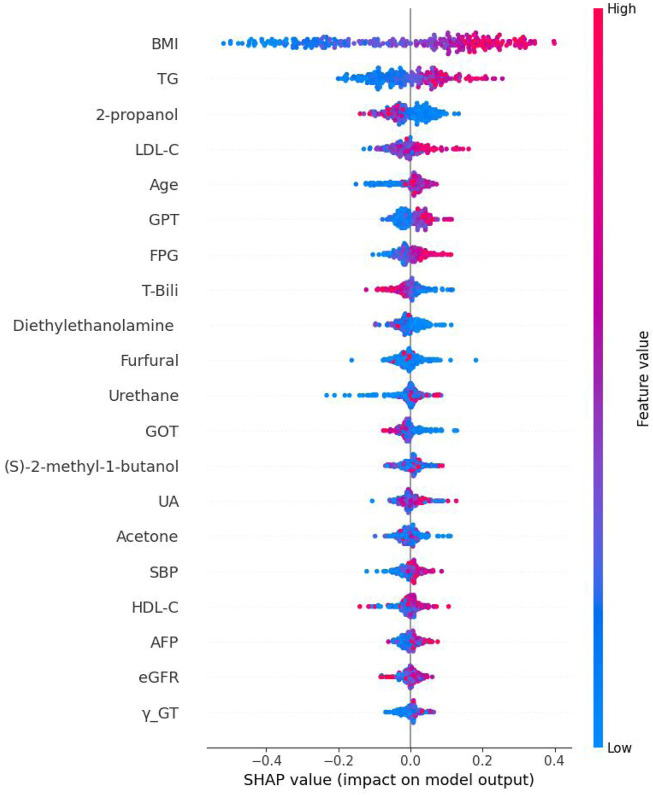
The Bee Swarm plot derived from Shapley addictive explanation of eXtreme Gradient Boosting. Note: BMI: body mass index; TG, Triglycerides; LDL-C, Low density lipoprotein cholesterol; GPT, Serum glutamic pyruvic transaminase; FPG, Fasting plasma glucose; T-Bili, Total bilirubin; GOT, Serum glutamic oxaloacetic transaminase; UA, Uric acid; SBP, Systolic blood pressure; HDL-C, High density lipoprotein cholesterol; AFP, Alpha-fetoprotein; eGFR, estimated Glomerular filtration rate; γ-GT, Gamma glutamyl transpeptidase.

**FIGURE 6:**
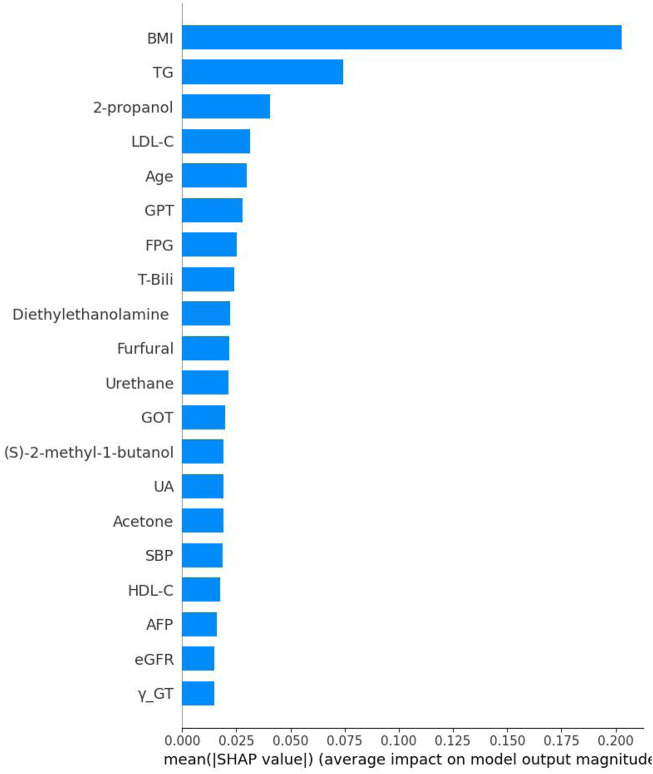
The absolute Shapley addictive explanation values of each feature. Note: BMI, body mass index; TG, Triglycerides; LDL-C, Low density lipoprotein cholesterol; GPT, Serum glutamic pyruvic transaminase; FPG, Fasting plasma glucose; T-Bili, Total bilirubin; GOT, Serum glutamic oxaloacetic transaminase; UA, Uric acid; SBP, Systolic blood pressure; HDL-C, High density lipoprotein cholesterol; AFP, Alpha-fetoprotein; eGFR, estimated Glomerular filtration rate; γ-GT, Gamma glutamyl transpeptid.

The Bee Swarm plot derived from the XGBoost SHAP was shown in Figure 5. From top to the bottom listed the features selected and the higher horizontal feature indicates it is more important. Each circle represents a participant’s value impact of that feature. The red color has stronger impact whilst the blue one has less. Thus, this figure shows the direction of impact of each participants. Finally, Figure 6 shows the absolute strengths each feature from the highest to the lowest.

## Discussion

To the best of our knowledge, this study represents the largest cohort to date in the field of breath-based diagnostics for NAFLD, with 1,501 participants included. Previous related studies typically involved fewer than 100 subjects (Grewal and Mahmood, 2009; Chen et al., 2015), thereby limiting the generalizability and statistical power of their findings. Additionally, those studies primarily employed traditional statistical methods, which often fail to capture non-linear relationships among complex variables. In contrast, our study applied 10 different Mach-L algorithms, demonstrating that the inclusion of 341 VOCs in Model 1 led to a notable improvement in AUC, ranging from 5.20% to 9.80%, across different modeling approaches.

Volatile organic compounds (VOCs)—produced through endogenous metabolism, microbiota activity, and various cellular processes—hold significant potential as non-invasive biomarkers for disease detection. One of the greatest strengths of VOC-based diagnostics lies in their non-invasive nature, making them ideal for monitoring chronic conditions, tracking disease progression, and conducting large-scale population screening where invasive procedures are impractical. Furthermore, VOCs may offer early indicators of disease, enabling prompt diagnosis and intervention. This is particularly crucial for diseases like cancer and metabolic disorders, where early detection significantly enhances treatment outcomes.

By constructing a quantitative VOCs library from both sub-healthy and diseased individuals, predictive models and diagnostic algorithms can be refined to detect diseases at earlier stages. Importantly, the combination of VOC data with traditional clinical parameters and biomarkers enhances the accuracy and robustness of predictive models. Thus, the integration of quantitative VOC analysis has great potential to advance preventive medicine and revolutionize early disease detection.

Nevertheless, for VOCs to be effectively implemented in clinical practice, further research and validation are essential. Our present study lays a solid foundation for enhancing non-invasive prediction of NAFLD, and additional studies based on these findings are currently underway. Compared to previous research utilizing breathomics in NAFLD patients (Table 6) (Akesson, 1977; Alkhouri et al., 2014; Calabrese et al., 2023), our study offers a more comprehensive investigation, not only due to its larger sample size, but also through the simultaneous consideration of VOCs and clinical data, and the application of multiple machine learning algorithms for predictive modeling—an approach not previously explored in this field. However, it should be noted that, at this stage, due to two reasons the application of VOC in clinical practice is not practical; first, the sensitivity and specificity are not high enough; second, the cost of VOCs is still high.

**TABLE 6:** Analysis pipelines of studies using breath-based VOCs towards non-alcoholic fatty liver prediction.

Technique	Sample size	# Of VOCs	Methods	References
GC-MS	46	127	Partial least square discriminant analysis	Calabrese et al., 2023
SIFT-MS	60	14	Canonical discriminant analysis	Alkhouri et al., 2014
GC-MS	60	220	Univariate analysis	Raman et al., 2013

In the present study we did use XGBoost in order to examine the directions and impacts of each variable. The interpretation was given in the results section and, out of the 20 features, 6 different VOCs were selected and 2-propanol was the third important VOCS.

From the initial 341 VOCs analyzed, the top 10 most relevant compounds were identified using machine learning algorithms. Ranked by importance, these VOCs were: 2-propanol, acetone, butyl 2-methylbutanoate, diethylethanolamine, urethane, β-caryophyllene, furfural, tridecane, 4-methyloctanoic acid, and (S)-2-methyl-1-butanol.

The gold standard for diagnosing NAFLD remains liver biopsy (Brunt et al., 1999; Kleiner et al., 2005), yet this approach is invasive and carries a complication risk of approximately 0.5% (Bravo et al., 2001; Piccinino et al., 1986). Alternative, less invasive methods such as the Fibrosis-4 Index, which incorporates age, liver enzymes, and platelet count, have shown a positive predictive value (PPV) of around 80% (Author Anonymous, 2025). Likewise, ultrasound has demonstrated high sensitivity and specificity (84.8% and 93.6%, respectively) in detecting moderate to severe steatosis. Given this, one might argue that VOC analysis is more labor-intensive and costly. However, the primary value of VOCs lies in their potential to uncover novel insights into NAFLD pathophysiology, which could eventually lead to new therapeutic targets. Importantly, this study did not establish a causal relationship between VOCs and NAFLD.

As expected, the top-ranking variables in our analysis were traditional risk factors, including BMI, triglycerides (TG), fasting plasma glucose (FPG), among others. The first VOC to appear in the list was 2-propanol. The well-established impact of traditional clinical variables should not be overlooked (Huh et al., 2022), but it must also be recognized that these factors may confound the identification of VOCs truly associated with NAFLD. To address this, our machine learning models adjusted for the effects of conventional predictors, allowing for a more accurate evaluation of VOC contributions.

Below is a brief discussion of the top 10 VOCs identified:

### 2-Propanol

Lu et al. demonstrated that subchronic exposure to 2-propanol in mice induced NAFLD through dysregulation of the AMPK signaling pathway (Lu et al., 2015). Interestingly, in our study, 2- propanol levels were lower in NAFLD subjects, suggesting possible upregulation of AMPK as a protective or compensatory modality (Fang et al., 2022). However, the specific mechanistic studies on 2-propanol and fatty liver are limited, its known hepatotoxicity and the findings from animal studies support the possibility that 2-propanol exposure can contribute to fatty liver development, especially with high or prolonged exposure (Satapathy et al., 2015; World HealthOrganizatiom-INTERNATIONAL P ROGRAMME ON CHEMICALSAFETY, 1990).

### Acetone

Solga et al. previously reported that breath acetone was associated with NAFLD in morbidly obese patients undergoing bariatric surgery (Solga et al., 2006). This may reflect decreased d-3- hydroxybutyrate dehydrogenase activity or altered NADH levels, leading to acetone accumulation. There might be two mechanisms behind this relationship. First, enhancing ketogenesis reduces hepatic lipid accumulation in preclinical models. Exogenous ketones (e.g., β-hydroxybutyrate) show anti-inflammatory and antifibrotic effects, suggesting protective roles (Kwon et al., 2024). Second, while elevated acetone may indicate metabolic stress in early NAFLD, targeted ketone supplementation or ketogenic diets could mitigate steatosis and inflammation in specific contexts (Kwon et al., 2024). Our findings were consistent, with higher acetone levels in NAFLD subjects, though the difference was not statistically significant.

### Butyl 2-methylbutanoate

This fatty acid ester, found naturally in apricots (Prunus armeniaca), has been associated with celiac disease and IBS (pubchem, 2025). Only one prior study has investigated its link to NAFLD, finding higher prevalence in NAFLD patients (Raman et al., 2013). It might have influences on NAFLD due to the gut microbiota alterations which correlates with elevated 2-butanone (a structurally related ketone), hinting at broader metabolic disruptions involving ester-like compounds (Del et al., 2016). In the same time, methyl tert-butyl ether, another ether compound, shows epidemiological links to NAFLD risk in humans, suggesting potential shared mechanisms for ester/ether-induced metabolic dysfunction (Cui et al., 2024). Additional research is needed to elucidate its pathophysiological role.

### Diethylethanolamine

Akesson reported that diethylethanolamine promotes the conversion of phosphatidylethanolamine to phosphatidylcholine, a hepatoprotective compound (Akesson, 1977). However, at present, there is no direct evidence linking it to NAFLD. It is well known that alcohol metabolism produces acetaldehyde and reactive oxygen species that cause fatty liver. While diethylethanolamine is not an alcohol, its metabolism might theoretically produce reactive intermediates that could similarly affect hepatic cells (Liu, 2014). Our findings—lower levels in NAFLD subjects—support this mechanism and suggest its potential protective role.

### Urethane

Studies in rats demonstrate that administration of carcinogenic doses of urethane leads to liver microsomal damage, including degranulation of liver microsomes, which impairs liver cell function and contributes to hepatic injury (Dani, 1983). In human, it is found that liver injury in workers exposed to N,N-dimethylformamide (DMF) (Nakasone et al., 2011; Nomiyama et al., 2001; Redlich et al., 1988), urethane levels were higher in the control group in our study. While this may indicate resistance to hepatic injury, further evidence is required to substantiate this hypothesis.

### β-caryophyllene

This anti-inflammatory, plant-derived compound activates CB2 receptors, reducing oxidative stress and hepatic injury in mice models (Varga et al., 2018). In the same time, it could reduce intracellular lipid accumulation, primarily by lower saturated fatty acids and modifying the lipid profile toward less harmful species (Scandiffio et al., 2023). Our findings align with this, supporting its potential therapeutic use.

### Furfural

Interestingly, furfural has a complex relationship with liver health. In low dose, it could improve mitochondrial function, reduced reactive oxygen species, and restoration of the NAD^+^/NADH redox balance, which is crucial for lipid metabolism and preventing fatty liver progression (Cheng et al., 2022). But when it is in high dose, a Maillard reaction product with antioxidant properties, furfural has demonstrated hepatocyte-protective effects in animal studies (Powell et al., 2014). This may explain its relevance in our NAFLD model.

### Tridecane

There is currently no direct evidence or well-established research linking tridecane specifically to NAFLD or its progression. Tridecane is a hydrocarbon (alkane) commonly found in petroleum products and some environmental pollutants, but its direct impact on liver fat accumulation or liver metabolism has not been clearly documented. The only evidence is that tridecane has been associated with inflammation and lipid peroxidation, particularly in distinguishing NASH from non-NASH (The good scents company, 2025).

### 4-methyloctanoic acid

Although largely known for its use in food flavoring, this compound is a fatty acid and may reflect metabolic changes. The possible mechanisms include it is a BCFA involved in lipid metabolism; it can modulate gene expression related to fatty acid metabolism; and there is an indirect link to the NAVLD (Zhao et al., 2022; Liu et al., 2018; Pooya et al., 2012). We observed higher levels in non-NAFLD subjects, potentially due to better hepatic metabolism in healthier individuals (Yamaguchi et al., 2007).

### (S)-2-methyl-1-butanol

Produced by *Saccharomyces cerevisiae*, this compound has antioxidant properties (Wilson et al., 2022; Agarwal et al., 2020; Landolfo et al., 2008), which may be linked to liver protection. In the same time, it is a fatty alcohol lipid molecule involved in metabolic pathways related to fatty acid and alcohol metabolism. It can be oxidized to 2-methylbutyrate, which then enters beta-oxidation to produce acetyl-CoA and propionyl-CoA, key intermediates in energy metabolism (Thompson et al., 2020). This indicates that (S)-2-methyl-1-butanol is metabolically linked to fatty acid catabolism through its conversion to fatty acid derivatives that feed into mitochondrial energy pathways. While no prior study has evaluated its relationship with NAFLD, our findings suggest it may play a role in hepatic defense mechanisms.

### Limitations

Despite the strengths of our study—including a large sample size and comprehensive VOC profiling—there are important limitations. In the present study, we do have limitations. First, this is a cross-sectional study which is less persuasive than a longitudinal one. There is no conclusion of cause-effect relationship could be drawn. However, since some of these participants will continue to have a health check up in our clinic, in the future, we believe that we will have a longitudinal study. Second, the participants were only limited to Taiwanese. It should be exercised to another ethnic group with cautious. In the future, since these participants will remain to be followed up in the MJ clinic, longitudinal studies could be done by using the present results of VOCs to predict future diseases. In the same time, we will separate these participants into two groups; one for selecting the VOCs, the rest will be treated as a validation group.

## Conclusion

Using 10 different Mach-L algorithms, we identified the relative importance of both clinical parameters and volatile organic compounds (VOCs) in predicting non-alcoholic fatty liver disease (NAFLD) within a cohort of 1,501 participants. Among clinical variables, the most influential features included body mass index (BMI), triglycerides (TG), uric acid (UA), fasting plasma glucose (FPG), γ-glutamyltransferase (γ-GT), gender, low-density lipoprotein cholesterol (LDL-C), and sleep duration.

In addition to traditional clinical predictors, 2-propanol emerged as the most influential VOC, followed in descending order by acetone, butyl 2-methylbutanoate, diethylethanolamine, urethane, β-caryophyllene, furfural, tridecane, 4-methyloctanoic acid, and (S)-2-methyl-1- butanol. The potential biological relevance and mechanisms of action of these VOCs were discussed in relation to liver metabolism and disease pathology.

While this study offers new insights into the role of VOCs as non-invasive biomarkers for NAFLD, its cross-sectional design limits the ability to determine causality. Future research should aim to conduct longitudinal studies to further elucidate the cause-effect relationships between VOCs and the development or progression of NAFLD.

## Data Availability

The raw data supporting the conclusions of this article will be made available by the authors, upon reasonable request.
